# Dietary vitamin D intake and vitamin D related genetic polymorphisms are not associated with gastric cancer in a hospital-based case-control study in Korea

**DOI:** 10.7555/JBR.32.20170089

**Published:** 2018-01-03

**Authors:** Sang-Yong Eom, Dong-Hyuk Yim, Dae-Hoon Kim, Hyo-Yung Yun, Young-Jin Song, Sei-Jin Youn, Taisun Hyun, Joo-Seung Park, Byung Sik Kim, Yong-Dae Kim, Heon Kim

**Affiliations:** 1 Departments of Preventive Medicine and Medical Research Institute, Chungbuk National University, Cheongju 28644, Korea; 2 Departments of Surgery, Chungbuk National University, Cheongju 28644, Korea; 3 Internal Medicine, College of Medicine, Chungbuk National University, Cheongju 28644, Korea; 4 Department of Food and Nutrition, Chungbuk National University, Cheongju 28644, Korea; 5 Department of Surgery, College of Medicine, Eulji University, Daejon 301746, Korea; 6 Department of Surgery, Asan Medical Center, College of Medicine, Ulsan University, Seoul 138736, Korea.

**Keywords:** gastric cancer, vitamin D, vitamin D receptor, gene-environment interaction

## Abstract

There have been few studies on the association between vitamin D levels and gastric cancer in Asian populations, but no studies have been performed on the interactions between vitamin D intake and polymorphisms in the vitamin D pathway. The effects of vitamin D intake, vitamin D related genetic polymorphisms, and their association with the incidence of gastric cancer were investigated in a hospital case-control study, including 715 pairs of newly diagnosed gastric cancer patients and controls matched for age and sex. Correlations between vitamin D intake and plasma vitamin D concentrations were also assessed in a subset of subjects. No statistically significant difference was observed in the dietary intake of vitamin D between the patients and controls, nor were there any evident associations between vitamin D intake and risk of gastric cancer in multivariate analyses. Vitamin D intake significantly correlated with the circulating 25-hydroxyvitamin D levels, but not with the active form of the vitamin, 1,25-dihydroxyvitamin D. There were no statistically significant interactions between vitamin D intake, and *VDR* or* TXNIP *polymorphisms. This study suggests that dietary vitamin D intake is not associated with gastric cancer risk, and the genetic polymorphisms of vitamin D-related genes do not modulate the effect of vitamin D with respect to gastric carcinogenesis.

## Introduction

Despite a decline in the incidence of gastric cancer worldwide, it is the second most common cancer and the third most common cause of cancer-related deaths in the Korean population^[1]^. Gastric cancer is particularly prevalent in East Asia, and it is associated with high rates of *Helicobacter pylori* (HP) infection, and dietary, environmental, and genetic risk factors, as well as the interaction between these factors^[2]^. HP infection and high salt intake lead to chronic active inflammation in the gastric epithelium, and chronic inflammation triggers atrophic gastritis and intestinal metaplasia, which are considered as precursor lesions of gastric cancer^[3]^. Additionally, intestinal metaplasia may progress to dysplasia and eventually develop into gastric adenocarcinoma^[4]^.

Vitamin D is involved in the anti-cancer and anti-inflammatory actions in the body^[5]^, and many epidemiological studies have reported a negative or positive association between vitamin D and various cancers, such as colon, breast, and prostate^[6^‒^9]^. Vitamin D is ingested *via* food (*e.g.*, milk, fish, mushrooms, *etc*.) and supplements, and is synthesized in the skin by sunlight exposure^[9]^. Vitamin D is converted into 25-hydroxyvitamin D (25-OH-D, calcidiol) in the liver, which is converted into 1,25-dihydroxyvitamin D (1,25-(OH)_2_-D, calcitriol) in the kidney^[5^,^10]^.

The antitumor activity of vitamin D is associated with its active form, 1,25-(OH)_2_-D, which is potent steroid hormone^[9^,^11]^. 1,25-(OH)_2_-D binds to vitamin D Receptors (VDR) and regulates the transcription of hundreds of genes harboring vitamin D-response elements, thereby mediating its effects on cell cycle, proliferation, differentiation, and apoptosis. A recent study demonstrated that 1,25-(OH)_2_-D suppresses the growth of gastric cancer cells in a VDR-independent manner *via* the mutant p53-mediated stabilization of VDR^[11]^. Vitamin D status in the body can be determined by polymorphisms of various vitamin D pathway genes including* VDR*^[7]^.

Although many studies have investigated the relationship between vitamin D and the development of cancer, the results have been inconsistent^[9]^. Moreover, while there have been few studies on vitamin D and the incidence of gastric cancer in Asian populations, to date no study has investigated the effects of vitamin D intake and polymorphisms in the vitamin D related genes, together. In this study, we determined the role of vitamin D in the development of gastric cancer by evaluating the effects of vitamin D intake, vitamin D related genetic polymorphisms, and the interaction of these factors in the incidence of gastric cancer.

## Materials and methods

### Study subjects

Study subjects included 715 patients newly diagnosed as having gastric cancer along with 715 age-matched and sex-matched control participants. Specific methods of the study have been previously described^[12^‒^13]^. Briefly, the study protocol was approved by the Institutional Review Board (CBNU-IRB-2012-BG01). Informed consent and information on lifestyle, particularly diet, was obtained from all the subjects. Dietary habits were assessed using a validated and semi-quantitative food frequency questionnaire (FFQ)^[14]^. Peripheral blood samples were collected from all the subjects for genotyping.

### Estimation of vitamin D intake and measurements of plasma vitamin D levels

The intake of vitamin D was estimated from the FFQ data, and the vitamin D content of each food item was referenced to the Korean Standard Food Composition Table^[15]^. The vitamin D intake for each food item was calculated by multiplying the food intake amount by the vitamin D content. The estimated weekly total intake of vitamin D was calculated by summing the amount of vitamin D intake for all food items. Plasma 25-OH-D and 1,25-(OH)_2_-D levels were measured in 72 patients and 91 control-participants from whom sufficient plasma could be collected. Plasma 25-OH-D and 1,25-(OH)_2_-D levels were measured using commercial enzyme-linked immunosorbent assay (ELISA) kits (25-OH vitamin D ELISA or 1,25 dihydroxyvitamin D EIA; ALPCO, Ltd., Windham, NH, USA) according to the manufacturer’s instructions.

### Genotyping analysis

Genomic DNA was isolated from peripheral blood using a DNA purification kit (DNA Extractor WB; Wako, Osaka, Japan) according to the manufacturer’s protocol. The single nucleotide polymorphisms (SNPs) of vitamin D-pathway genes, including, *VDR* (rs1544410, rs2239179, and rs4516035) and thioredoxin-interacting protein (*TXNIP* rs7211) were analyzed using the GoldenGate assay. All polymorphisms satisfied the Hardy-Weinberg equilibrium.

### Statistical analysis

A Student’s *t* test was used to compare continuous variables (*e.g.*, the body mass index, dietary vitamin D intake, and plasma vitamin D level) among the patients and control subjects. The chi-square test was used to analyze categorical variables (*e.g.*, smoking status, drinking status, education level, and HP infection) and categorized variables (*e.g.*, the cumulative amount of smoking and vitamin D intake). Correlations between the vitamin D intake and plasma vitamin D levels were evaluated using the Spearman correlation coefficient. The effects of vitamin D intake and the SNPs on gastric cancer incidence were assessed by multivariate conditional logistic regression analysis; the covariates included smoking, alcohol consumption, education level, HP infection, and total caloric intake. The weekly dietary vitamin D intake was categorized by its tertile among the control subjects. A stratified analysis was used to estimate the potential joint effects between the genotypes and the combined effects of those genotypes and the vitamin D intake. Statistical analyses were performed using SAS software (version 9.4; SAS Institute, Cary, NC, USA).

## Results

There were higher proportions of smokers and HP-infected individuals among the patients than in the control group; however, the level of education was higher in the control group than in the patients. The vitamin D intake was lower in patients [(42.9±38.3) µg/week] than in the controls [(46.0±38.1) µg/week]. However, this difference was not significant. When the vitamin D intake was divided into three groups based on tertiles in the control group, the patient group was the most distributed in the lowest tertile group (37.3%) and the least distributed in the highest tertile group (30.8%). However, statistical significance of the associations between vitamin D intake and the risk of gastric cancer was not observed. The mean plasma concentration of 25-OH-D was significantly lower in patients [(17.1± 8.9) ng/mL] than in controls [(20.0±6.5) ng/mL]. Plasma 1,25-(OH)_2_-D levels were also significantly lower in patients [(45.9±20.3) pg/mL] than in controls [(56.2±23.6) pg/mL, ***Table 1***]. Vitamin D intake was significantly correlated with plasma 25-OH-D levels (patients: *r*=0.277 and *P*<0.05; controls: *r*=0.271 and *P*<0.01), but this was not so for plasma 1,25-(OH)_2_-D levels (patients: *r*=0.018 and *P*=0.882; controls: *r*=0.051 and *P*=0.629) (***Fig*** ***.*** ***1***). There was no significant association between* VDR* and *TXNIP* polymorphisms and the risk of gastric cancer. There were no statistically significant interactions between vitamin D intake, and the *VDR* or *TXNIP* polymorphisms (***Table 2***).

**Tab.1:** General characteristics of the study population

Variables	Patients	Controls	*P* value or odds ratio (95% CI)
Total (*n*)	715	715	
Age (mean±SD), years	57.1±10.8	56.9±11.0	0.732
Sex [*n*(%)]			
Male	455 (63.6)	455 (63.6)	1.00 (reference)
Female	260 (36.4)	260 (36.4)	‒
Smoking status [*n*(%)]			
Nonsmokers	296 (41.4)	318 (44.5)	1.00 (reference)
Smokers	419 (58.6)	397 (55.5)	1.38 (0.99‒1.93)
Cumulative smoking amount [*n*(%)]			
Nonsmokers	296 (41.4)	317 (44.3)	1.00 (reference)
0‒19 pack-years	95 (13.3)	108 (15.1)	1.16 (0.77‒1.74)
20‒39 pack-years	176 (24.6)	166 (23.2)	1.46 (0.99‒2.14)
>40 pack-years	148 (20.7)	124 (17.3)	1.57 (1.05‒2.37)
Alcohol intake status [*n*(%)]			
Nondrinkers	278 (38.9)	276 (38.6)	1.00 (reference)
Drinkers	437 (61.1)	439 (61.4)	0.94 (0.71‒1.24)
Education level [*n*(%)]			
<High school	423 (60.9)	311 (43.9)	1.00 (reference)
≥High school	272 (39.1)	398 (56.1)	0.34 (0.25‒0.46)
*H. pylori* infection [*n*(%)]			
Negative	160 (22.4)	214 (29.9)	1.00 (reference)
Positive	555 (77.6)	501 (70.1)	1.31 (1.01‒1.68)
Body mass index (BMI), kg/m^2^	22.2±3.3	23.5±3.0	<0.001
Dietary vitamin D intake (µg/week)	42.9±38.3	46.0±38.1	0.124
Tertile 1,<22.9 [*n*(%)]	267 (37.3)	239 (33.4)	1.00 (reference)
Tertile 2, 22.9-53.4 [*n*(%)]	228 (31.9)	238 (33.3)	0.93 (0.71‒1.21)
Tertile 3,>53.4 [*n*(%)]	220 (30.8)	238 (33.3)	0.94 (0.71‒1.23)
Plasma 25-(OH)-vitamin D (ng/mL)	17.1±8.9	20.0±6.5	0.024
Plasma 1,25-(OH)_2_-vitamin D (pg/mL)	45.9±20.3	56.2±23.6	0.004

CI: confidence interval

**Fig.1:**
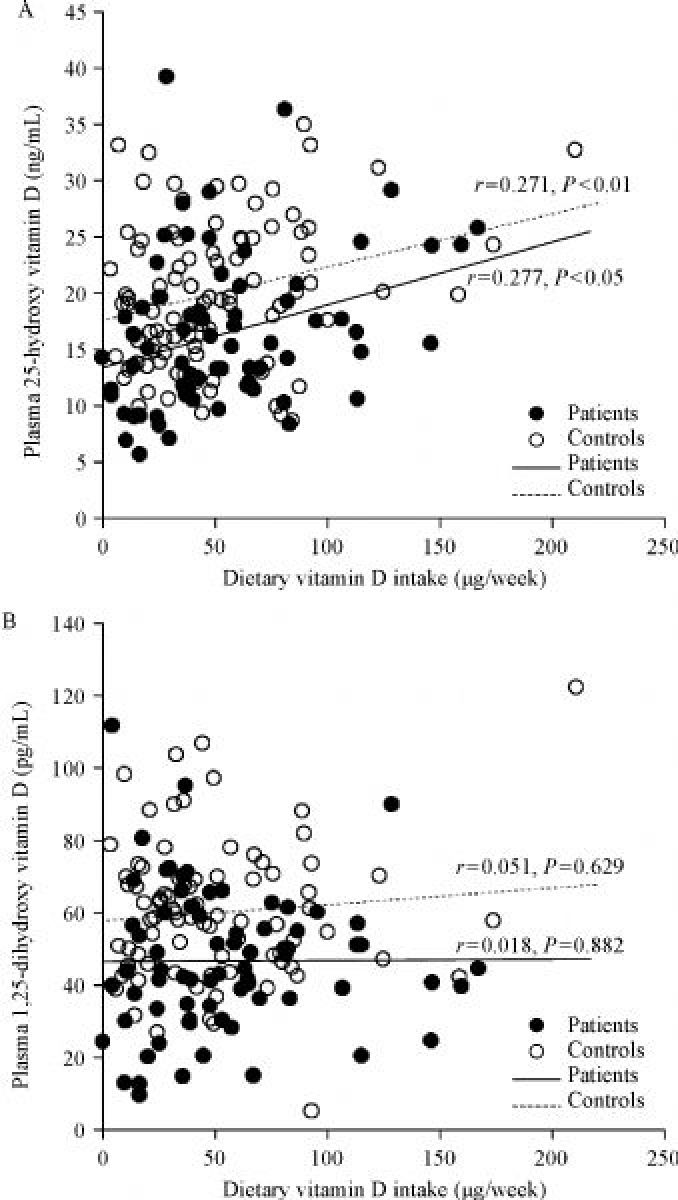
Correlation of plasma 25-hydroxyvitamin D (A) and 1,25-dihydroxyvitamin D (B) with dietary vitamin D intake in patients with gastric cancer and the controls

**Tab.2:** Influence of the interactions between dietary vitamin D intake and the four genetic polymorphisms on the risk of gastric cancer

Genetic polymorphisms		All	Dietary vitamin D intake (µg/week)			
			Tertile 1,<22.9	Tertile 2, 22.9-53.4	Tertile 3,>53.4	*P*_interaction_
VDR rs1544410	GG GA/AA	1.00 (reference) 1.06 (0.65‒1.75)	1.00 (reference) 0.83 (0.38‒1.82)	0.86 (0.57‒1.29) 1.05 (0.38‒2.95)	0.95 (0.61‒1.46) 1.17 (0.51‒2.67)	0.5372
VDR rs2239179	AA AG/GG	1.00 (reference) 1.10 (0.81‒1.50)	1.00 (reference) 1.39 (0.85‒2.28)	1.21 (0.74‒1.99) 0.82 (0.47‒1.41)	1.04 (0.62‒1.72) 1.39 (0.79‒2.46)	0.6812
VDR rs4516035	TT TC/CC	1.00 (reference) 0.86 (0.39‒1.90)	1.00 (reference) 0.76 (0.25‒2.30)	0.88 (0.59‒1.32) 0.69 (0.14‒3.30)	1.01 (0.67‒1.53) 1.23 (0.23‒6.56)	0.6818
TXNIP rs7211	CC CT/TT	1.00 (reference) 0.92 (0.74‒1.15)	1.00 (reference) 1.02 (0.69‒1.50)	1.16 (0.81‒1.67) 1.12 (0.75‒1.67)	1.38 (0.95‒2.01) 1.00 (0.66‒1.52)	0.1952

The data are presented as odds ratio (95% confidence interval) adjusted for age, sex, smoking, drinking, education level, *H. pylori* infection, and total caloric intake. A: adenine; C: cytosine; G: guanine; T: thiamine; TXNIP: thioredoxin-interacting protein; VDR: vitamin D receptor.

## Discussion

This study evaluated the relationship between vitamin D intake, vitamin D related polymorphisms, and the risk of gastric cancer in an Asian population. We did not observe any significant associations between vitamin D intake, and the risk of gastric cancer in Koreans; furthermore, vitamin D related genetic polymorphisms did not affect these associations.

Although there was no statistical significance, the vitamin D intake in patients with gastric cancer was lower than that of the control group by about 3 µg/week. The risk of gastric cancer in the middle and highest vitamin D intake tertile groups was lower at 6%‒7% than in the lowest tertile group. In addition, plasma vitamin D levels were significantly lower in patients with gastric cancer than in controls. This consistent result shows the potential for the anticancer effect of vitamin D, although it is weak and cannot reach significance. As with our findings, a meta-analysis evaluating the effects of vitamin D on the risk of gastric cancer found no association between the vitamin D intake and risk of gastric cancer; likewise, no significant association was found between plasma 25-OH-D levels and the risk of gastric cancer^[16]^. However, anti-cancer effects of vitamin D on gastric carcinogenesis have been reported continuously in experimental studies^[11^,^17]^. A recent retrospective chart analysis reported that vitamin D deficiency was related to an increased risk of gastric cancer^[18]^. One of the reasons for the heterogeneity of these studies may be the failure to accurately assess the association between vitamin D status and anticancer effects.

Many epidemiological studies have used plasma or serum 25-OH-D concentrations as a biological marker for assessing the vitamin D status^[7^‒^8^,^19]^. Although 25-OH-D reflects the body’s vitamin D status through dietary intake and body synthesis, it has a seasonal variation and is affected by calcium concentration in the body and the parathyroid hormone^[20]^. In addition, the 25-OH-D level in patients after diagnosis reflects changes in diet and lifestyle, a decrease of sun exposure due to hospitalization, and cancer treatment^[19]^. Since the 25-OH-D level in blood of subjects of case-control studies could be distorted for those reasons, it is more appropriate to use the estimated vitamin D intake level as a surrogate for the vitamin D status in the body. In this study, we observed a significant correlation between the vitamin D intake based on FFQ data and plasma 25-OH-D levels in patients with gastric cancer and the controls. Similar to our results, the Cohort Consortium Vitamin D Pooling Project of Rarer Cancer also confirmed a moderate correlation between the total vitamin D intake and serum concentration of vitamin D (*r*=0.26, *P*<0.001)^[21]^. This suggests that the vitamin D intake using dietary data is valid and reflects the actual vitamin D levels in the body. However, in the present study, the vitamin D intake did not correlate with the plasma level of 1,25-(OH)_2_-D, which is the biologically active form of vitamin D that downregulates cellular proliferation^[5^,^9]^. In other words, vitamin D intake measured in this study itself does not represent the degree of biologically active anticancer activity. Therefore, in order to evaluate the anticancer activity of vitamin D, genetic polymorphisms related to vitamin D active form conversion should be considered.

VDR is a steroid hormone receptor that binds to 1,25-(OH)_2_-D and mediates the various antineoplastic actions of vitamin D^[9]^. The deficiency of VDR is associated with an increased cancer incidence, and there are about 200 SNPs^[5]^. However, the three *VDR* SNPs (rs1544410, rs2239179, and rs4516035) investigated in this study were not associated with a risk of gastric cancer. Gene-environmental interaction between the vitamin D intake and *VDR *SNP also did not affect the gastric cancer risk. To our knowledge, only one study has evaluated *VDR* SNP and the incidence of gastric cancer, and reported that the *VDR* rs10735810 gene polymorphism was associated with gastric cancer susceptibility^[22]^. Similar to our results, 33 *VDR* SNPs were not associated with colorectal cancer development in Japanese patients^[23]^. Ashmore *et al.* did not observe genetic‒environmental interactions on vitamin D intake and *VDR* SNPs in colorectal cancer either^[24]^.

In this study, *TXNIP* genetic variation was also evaluated with *VDR* SNPs. TXNIP is a protein that binds to thioredoxin and is associated with oxidative stress and immune responses^[25]^. It is upregulated by 1,25-(OH)_2_-D. A previous study showed that TXNIP inhibits HP-associated gastric cancer by inhibiting the induction of tumor necrosis factor-alpha, nuclear transcription factor-kappa B, and cyclooxygenase-2^[26]^. In addition, an increased incidence of HP-induced gastric cancer was reported in *TXNIP* knockout mice, and *TXNIP* mRNA expression in gastric cancer tissues of patients was significantly lower than that in surrounding normal tissues^[26^‒^27]^. Despite the biological relevance of TXNIP in gastric carcinogenesis, we could find no significant association between the genetic variation of *TXNIP* and gastric cancer in the present study. These results suggest that VDR and TXNIP may play an important role in the progression of gastric cancer, but not in the initiation.

This study has some limitations. First, because this study was performed with a case-control study design, it carries an inherent information bias. However, to overcome this bias, we selected incident cases and assessed vitamin D intake based on the usual diet before gastric cancer was diagnosed. Additionally, the estimated vitamin D intake in this study does not fully reflect the total intake of vitamin D because it was estimated from the 89 food items included in the FFQ. In particular, vitamin D intake through supplements was not reflected in this study because supplemental intake information was not available. However, supplementation was closely related to age, sex, and socioeconomic status^[28]^, and these variables were included in the final multivariate model as covariates. Finally, only some of the gene polymorphisms associated with vitamin D were evaluated in this study. In particular, genetic variations in *cytochrome P450 27B1* (*CYP27B1*) and *CYP24A1* genes, which play a key role in vitamin D metabolism, were not evaluated. Therefore, large-scale prospective studies are needed to clarify the epidemiologic relationship between vitamin D and the risk of gastric cancer. In further study, it is necessary to investigate the SNPs involved in the production and degradation of 1,25-(OH)_2_-D in vitamin D metabolism.

In conclusion, we found no association between gastric cancer and vitamin D intake in this Korean population; we also did not find any genetic-environmental factor interactions between the vitamin D-related polymorphisms and vitamin D intake.
